# Predicators for Weight Gain in Children Treated for Severe Acute Malnutrition: A Prospective Study at Nutritional Rehabilitation Center

**DOI:** 10.1155/2014/808756

**Published:** 2014-03-12

**Authors:** Jyoti Sanghvi, Sudhir Mehta, Ravindra Kumar

**Affiliations:** ^1^Department of Pediatrics, Sri Aurobindo Medical College and PG Institute, Indore, Madhya Pradesh 453555, India; ^2^Central Research Laboratory, Sri Aurobindo Medical College and PG Institute, Indore, Madhya Pradesh 453555, India

## Abstract

*Introduction*. Despite being an important health problem in developing countries, there is little information available on factors affecting the severe acute malnutrition, especially nondietary factors. *Objective*. To study the impact of various factors, especially nondietary ones affecting directly or indirectly the weight gain in children with severe acute malnutrition. *Method*. A total of 300 children in the age group of 6 to 60 months meeting the WHO criteria for severe acute malnutrition were enrolled in the study. These children were provided special therapeutic diet as recommended by WHO/UNICEF protocol. Children were called for followup every 15 days up to 2 months after discharge to evaluate whether these children have achieved a final target weight gain of 15% of their admission weight. The impact of nondietary factors related to child, mother, and socioeconomic status was evaluated. Data collected through structured questionnaire were analyzed. *Result*. 172 (57.4%) of the total 300 children did not gain final target weight despite giving adequate diet. We observed that impact of various nondietary factors like mother's educational status and her knowledge about feeding practices, socioeconomic status, previous history, and present evidence of infection in child was important in determining the weight of child. No association was found with gender of child, BMI of mother, and father's educational status on the weight gain of child. *Conclusion*. The findings of this study confirm the association of many nondietary factors with weight gain in children treated for severe acute malnutrition. To reduce malnutrition emphasis should be given on these factors.

## 1. Introduction

Malnutrition remains a major public health problem throughout the developing world and is an underlying factor in over 50% of the children deaths under 5 years who die each year of preventable causes [1–4]. Approximately 9% of sub-Saharan African and 16% South Asian children suffer from moderate acute malnutrition and approximately 2% of children living in developing countries suffer from severe acute malnutrition [5, 6]. This is equivalent to approximately 60 million children suffering from moderate malnutrition and 13 million suffering from severe acute malnutrition at any one time. In India approximately 20% of children under five years, are severely wasted [7]. Estimates from most recent nationally representative survey indicate that 6.4% of children below 60 months of age have weight-for-height below third standard deviation. At Present Indian population of 1.2 billion, there are about 132 million children under five years (12% of population), of which 6.4% or roughly 8 million are assumed to be suffering from severe acute malnutrition. To prevent deaths among severely malnourished children identified under this drive, the Government of Madhya Pradesh, India, started the Nutrition Rehabilitation Centers (NRCs) under the Bal Shakti Yojna with support of UNICEF. The objectives of the programme are to control malnutrition among the children aged 1–5 years in the state and to bring down the percent of severely malnourished children to less than 1% [8]. This work was done on the basis of the WHO protocol for management of severe acute malnutrition. Therefore, malnutrition is an important public health problem in India. However, little information is available on risk factors for severe acute malnutrition (SAM), especially nondietary ones. Mother's formal education, nutrition, her knowledge about infant feeding practices, working status, family beliefs, socioeconomic status, and any underlying infections or illnesses from which the child may be suffering have impact on overall nutrition and weight gain.

In this study, we tried to determine influence of these risk factors other than diet which are related to child, mother, or socioeconomic status which could lead to SAM in children under the age of five.

## 2. Material and Methods

This was a prospective observational study done for a period of 15 months from October 2011 to December 2012 at SAIMS Hospital Indore, which is a tertiary care hospital having 20 bedded nutrition rehabilitation center (NRC), which runs with help of WHO/UNICEF. The study participants were 300 children of severe acute malnutrition between 6 and 60 months of age. These children were enrolled on the basis of WHO criteria for severe acute malnutrition, which included children with weight-for-height (*W*/*H*) or length (*W*/*L*) with *Z* score less than 3 standard deviation, and/or *W*/*H* or *W*/*L* with *Z* score less than 2 SD with mid-upper arm circumference (MUAC) <11.5 cm, and/or presence of bilateral pitting edema. The children who met this criterion were admitted to NRC and given special therapeutic diet including F 75 and F 100 as per WHO/UNICEF protocol for management of severe acute malnutrition (WHO 1999). These children are observed for weight gain of at least 5–10 gm/kg/day during hospital stay of 14 days. These children were finally discharged on day 14 and followed up to 2 months every 15 days to achieve the final target weight gain of 15% of the weight at the time of admission in NRC.

After ruling out the acute complications and their initial stabilization, these children were subjected to the actual NRC protocol. Children were given NRC diet as per WHO/UNICEF protocol daily along with other supplements. WHO weight for height reference charts was used for their assessment. Daily weight measurements were done at fixed time using a single standardized weighing scale provided by UNICEF and at the end of 14 days average weight gain in grams per kg per day was calculated. Weight gains more than 5 gm/kg/d were considered satisfactory. During hospital stay mothers were interviewed using detailed structured questionnaire and the impact of various nondietary factors related to child, mother, and socioeconomic status was recorded. On the 14th day children were discharged with dietary advice and multivitamins and iron supplements. The children were followed up every 15th day after discharge from hospital. At each visit anthropometric measurement was done. The final target was the weight gain of 15% of admission weight at the end of fourth followup. The children who finally gained 15% of weight at the time of admission were labeled as “cured” and who did not gain the target weight were labeled as “not cured” and finally constituted “weight gained” or “not weight gained” group, respectively. The comparisons were done between these two groups for various factors especially nondietary ones for their impact on malnutrition which included factors related to (I) child-birth weight, that is, SFD/AFD (small/appropriate for gestation or date), history of recurrent infections (more than 2 infections in a year) in the past, and evidence of infection at time of admission; infant-child feeding practices which included duration of exclusive breastfeeding, age of starting complementary feeding, birth order, any underlying medical or systemic illness; (II) study of factors related to mother such as mother's malnutrition which was assessed using mother's BMI, mother's education status, mother's working status, that is, working/housewife, and her knowledge about infant feeding practices; (III) socioeconomic status, which was assessed using modified Kuppuswamy's scale. All of the information was collected by mother's interviews using structured questionnaires. The data were analyzed by Chi square test using SPSS software.

## 3. Results

The study included 300 children, out of which 144 were males and the remaining 156 were females. The mean age was 22.95 ± 13.68 months: for males 24.29 ± 13.18 months and for females 22.75 ± 13.94 months. Overall families of 87.3% (262) of the study subjects belonged to poor socioeconomic status, that is, classes IV and V as per modified Kuppuswamy's socioeconomic scale. 30 (10%) of total children had underlying systemic/medical illness (12 (40%) CNS, 8 (26%) respiratory, 6 (20%) CVS, 2 (6%) GIT, and 2 (6%) metabolic). The incidence of tuberculosis was found to be 30 (10%) of total children and of these 26 (86.6%) achieved final target weight after treatment with ATT.

Despite giving adequate therapeutic diet at the end of 14 days, only 39.0% (117) children (51 boys and 66 girls) started gaining more than 5 gram/kg/day and only 128 (42.6%) children gained final target weight at the end of followup of 2 months, while 172 (57.4%) children did not gain final target weight. A statistically significant difference was observed between the mean weight at discharge after 14 days and the mean weight at admission for these 117 children, who had weight gain more than 5 gram/kg/day (*P* < 0.001). At end of study, two groups were formed, that is, weight gain (group I) and nonweight gain group (group II). Final comparisons were done between these two groups (172 with no weight gain and 128 with weight gain).

Table 1 shows the distribution pattern of age groups in two groups. We observed that children in higher age group did not gain weight as compared to children in lower age group. We did not find any significant difference in sex ratio in both the groups. Evidence of infection at admission was about two times higher in group II compared to group I (*P*  value = 0.0038, OR 1.66, 95% CI 1.027–2.686). There was history of recurrent infections in 14 and 34 children in groups I and II, respectively.

We did not find any correlation between mothers BMI and these two groups (20.17 ± 2.5 kg/m^2^ in group I and 20.34 ± 2.52 kg/m^2^ in group II, *P*  value = 0.547).

The illiteracy rate (i.e., no formal education or <5th standard) was significantly higher among mothers of group II than the mothers of group I (Table 2). We found no association of father's literacy rate among two groups. 139 (80.8%) mothers of group II were working compared to 91 (70.1%) of group I; difference was statistically significant; *P*  value = 0.049 (OR = 1.713, 95% CI 0.999–2.935). A larger family size with the number of children equal to or more than 3 was noticed more significantly in nonweight gain group than in weight gain group. 165 (95.9%) children belonged to low SES in group II compared to 97 (75%) children in weight gain group (*P*  value < 0.0001, OR 7.533, 95% CI 3.195–17.764).

### 3.1. Nutritional Practices

To begin with all the children in both the groups were breastfed. Late initiation of complementary diet (after 6 months, mean age 9 months) was also more common in group II (13.4%) than in group I (6.2%) (*P*  value = 0.039, OR = 1.638, 95% CI 1.025–2.617) which was statistically significant. Bottle feeding was more frequently used in nonweight gain group (33 (26.5%)) than in weight gain group, that is, 17 (13.1%) (OR = 1.550, 95% CI 0.824–2.929).

### 3.2. Parental (Caregivers') Knowledge on Infant and Young Child Nutrition

Most of the caregivers in group II did not have knowledge about feeding practices. 120 (93.8%) of mothers in group I knew that breastfeeding should be initiated within the first hour of birth compared to 149 (86.6%) of group II (*P*  value = 0.045, OR = 2.315, 95% CI 2.100–5.361) (Table 3). Only 41.9% of the caregivers in group II knew that complementary diet should be started at the age of 6 months compared to 55.5% in weight gain group (*P*  value = 0.020, OR = 1.730, 95% CI 1.090–2.745). 72.3% of mothers from group II knew the importance of exclusive breast feeding up to 6 months which is very low as compared to 86.7% in mothers from group I (*P*  value = 0.0039, OR = 1.915, 95% CI 1.027–3.569). Only 83 (48.3%) mothers knew the advantages of breastfeeding in group II compared to 77 (60.2%) in group I (*P*  value = 0.041, OR = 1.619, 95% CI = 1.019–2.573). 31 (18%) mothers in group II compared to 25 (19.5%) in group I used to wash hands before giving food to children; it was not statistically significant.

## 4. Discussion

Parental illiteracy especially maternal is found to be associated with a higher risk of SAM (severe acute malnutrition). A study done by Haidar et al. [9] in rural areas of North Wollo, Ethiopia, had found a significant risk of severe acute malnutrition associated with maternal illiteracy. Similar results were obtained in other previous studies [10–16].

In a case-control study done in Bangladesh by Sakisaka et al. 2006 [16] maternal illiteracy was associated with a fourfold increase in the risk of severe acute malnutrition in their children. Another similar case control study “Risk factors for clinical marasmus” done by Henry et al. [17] from Bangladesh found a significant association with maternal education and risk of malnutrition. The risk of SAM (severe acute malnutrition) is increased with poverty. Similarly poor family income and low socioeconomic status have been found as a risk factors for severe acute malnutrition in studies done by previously [9–11, 18–20].

The study “Risk factors for malnutrition among rural Nigerian children” done by Odunayo and Oyewole [18] found a significant relationship between poverty and incidence of malnutrition. Similarly Coulter et al. [19], Radebe et al. [20], Getaneh et al. [21], Jeyaseelan and Lakshman [10], and Aminul Islam et al. [11] found similar association between prevalence of malnutrition with socioeconomic factors and family background.

A larger family size or high birth order is associated with an increased risk of severe acute malnutrition. The effect of a large family size with overcrowding leading to poor hygiene finally to recurrent infections and inadequate spacing has been implicated as a risk factor for severe malnutrition in different studies. A similar relationship was observed in study done by Haidar et al. [9]. Ighogboja [12] and Odunayo and Oyewole [18] from Nigeria and Henry et al. [17] from Bangladesh also observed a similar relationship in their study.

This supports the effect of the above socioeconomic risk factors on SAM. However, the impact was less in magnitude in this study when compared to the effect of infant and young child feeding practices.

Breastfeeding is a norm in India; nearly all the children in both groups were breastfed. In both groups breastfeeding was initiated within the first hour of birth in 216 (72%) of these children. Introduction of top feeds before six months of age was 2.9 times more common in children in nonweight group than in weight gain group; and initiation of complementary diet late after 6 months of age was 4.2 times more common in nonweight group, indicating that children with severe acute malnutrition who were started with complementary diet either too early or too late responded less to therapeutic diet. The study “Feeding practices in 105 counties of rural China” done in China by Wang et al. [22] showed that the introduction of other diets before the age of six months increased the prevalence of pneumonia and diarrheal disease. Similarly a study in western Kenya by Bloss et al. [23] showed an increased risk of being underweight when complementary food was started early. As a global public health recommendation, infants should be exclusively breastfed for the first 6 months of life to achieve optimal growth, development, and health.

Thereafter to meet their evolving nutritional requirements, infants should receive nutritionally adequate and safe complementary foods while breastfeeding continues for up to two years of age or beyond. Bottle feeding is more commonly observed in nonweight gain group than the weight gain group. Bottle feeding is discouraged at any age. It is usually associated with increased risk of illness, and especially diarrheal disease, because of the difficulty in sterilizing the nipples. Bottle feeding also shortens the period of postpartum amenorrhea and increases the risk of pregnancy.

A statistically significant difference in knowledge of mothers about infant feeding practices, that is, on the recommended duration of breastfeeding and on the appropriate time of initiating complementary diet between nonweight gain group and weight gain group, was observed. Beghin [24] in his critical assessment of 21 NRCs across 6 Latin American countries also found nutrition education to be lacking at most of the centers. This indicates that it is not only lack or shortage of food that predisposes young children to malnutrition but also lack of knowledge on appropriate infant and young child feeding practices.

In conclusion this study confirms the association of severe acute malnutrition with inappropriate infant and young child feeding practices. Other than therapeutic diet, factors such as occurrence of recurrent infections, presence of systemic illness, and socioeconomic status play an important role in deciding the weight gain in children treated for SAM. To reduce childhood malnutrition due emphasis should be given in improving the knowledge and practice of mothers on appropriate infant and young child feeding practices. However, as this is a hospital-based study further community-based studies are recommended to identify risk factors for weight gain in severe acute malnutrition.

## Figures and Tables

**Table 1:** Demographic data of children in two groups.

Parameters	Group I	Group II	*P* value, OR (95% CI)
Sex			
Male	62	74	0.361, 1.244 (0.786–1.93)
Female	66	98	
Age groups (in months)			
6–12	48 (46.2%)	56 (53.8%)	0.041
13–24	66 (46.8%)	75 (53.2%)	
25–36	8 (27.6%)	21 (72.4%)	
37–48	6 (3%)	14 (70%)	
49–60	0 (0%)	69 (100%)	
E/O infection at time of admission			
Yes	40 (35.1%)	74 (64.9%)	0.0038, 1.66 (0.027–2.686)
No	88 (47.3%)	98 (52.7%)	
H/O Recurrent infections in past			
Yes	14 (10.9%)	34 (19.8%)	0.039, 0.498 (0.255–0.974)
No	114 (89.1%)	138 (80.2%)	
Birth order			
<3	40 (35.1%)	74 (64.9%)	0.045, 1.73 (1.009–2.966)
≥3	88 (47.3%)	98 (52.7%)	

**Table 2:** Parent's education/working status in two groups.

Parameter	Weight gain	Nonweight gain	*P* value, OR (95% CI)
Mother's education status			
Illiterate	34 (26.6%)	49 (28.5%)	0.024
<5th standard	28 (21.9%)	59 (34.3%)	
>5th standard	66 (51.6%)	647 (37.2%)	
Father's education status			
Illiterate	27 (21.9%)	27 (15.7%)	0.316
<5th standard	42 (32.8%)	69 (40.1%)	
>5th standard	59 (46.1%)	76 (44.2%)	
Mother's working status			
House wives	37 (28.9%)	33 (19.2%)	0.049, 1.713 (0.999–2.935)
Working	91 (71.1%)	139 (80.8%)	
Socioeconomic status			
Classes I–III	31 (25%)	7 (0.1%)	0.0001, 7.533, (3.195–17.764)
Classes IV and V	97 (75%)	165 (95.9%)	

**Table 3:** Mother's knowledge about feeding.

	Weight gain	Nonweight gain	*P* value, OR, (95% CI)
When to start breastfeeding after birth			
At birth	120 (93.8%)	149 (86.6%)	0.045, 2.315 (1.000–5.361)
Later on	8 (6.2%)	23 (13.4%)	
Continue exclusive breastfeeding up to 6 months			
Yes	111 (86.7%)	133 (72.3%)	0.039, 1.915 (1.027–3.569)
No	17 (13.3%)	39 (22.7%)	
When to start complementary feeding			
6 months	71 (55.5%)	72 (41.9%)	0.020, 1.730 (1.090–2.745)
Later on	57 (44.5%)	100 (58.1%)	
Advantage of breastfeeding			
Yes	77 (60.2%)	83 (48.3%)	0.041, 1.619 (1.019–2.573)
No	51 (39.8%)	89 (51.7%)	

## References

[1] Black RE, Morris SS, Bryce J (2003). Where and why are 10 million children dying every year?. *The Lancet*.

[2] Caulfield LE, de Onis M, Blössner M, Black RE (2004). Undernutrition as an underlying cause of child deaths associated with diarrhea, pneumonia, malaria, and measles. *The American Journal of Clinical Nutrition*.

[3] Rice AL, Sacco L, Hyder A, Black RE (2000). Malnutrition as an underlying cause of childhood deaths associated with infectious diseases in developing countries. *Bulletin of the World Health Organization*.

[4] Pelletier DL, Frongillo EA (2003). Changes in child survival are strongly associated with changes in malnutrition in developing countries. *Journal of Nutrition*.

[5] Unicef Nutritional Status. http://www.childinfo.org/malnutrition_nutritional_status.php.

[6] World Health Organization Global Database on Child Growth and Malnutrition. http://www.who.int/nutgrowthdb/estimates2012/en/.

[7] International Institute for Population 
Sciences (IIPS) and Macro International (2007). *National Family Health Survey (NFHS-3), 2005-06: India*.

[8] World Health Organization (1999). *Management of Severe Malnutrition: A Manual For Physicians 11 and Other Senior Health Workers*.

[9] Haidar J, Abate G, Kogi-Makau W, Sorensen P (2005). Risk factors for child under-nutrition with a human rights edge in rural villages of North Wollo, Ethiopia. *East African Medical Journal*.

[10] Jeyaseelan L, Lakshman M (1997). Risk factors for malnutrition in south Indian children. *Journal of Biosocial Science*.

[11] Aminul Islam M, Mujibur Rahman M, Mahalanabis D (1994). Maternal and socioeconomic factors and the risk of severe malnutrition in a child: a case-control study. *European Journal of Clinical Nutrition*.

[12] Ighogboja SI (1992). Some factors contributing to protein-energy malnutrition in the middle belt of Nigeria. *East African Medical Journal*.

[13] Appoh LY, Krekling S (2005). Maternal nutritional knowledge and child nutritional status in the Volta Region of Ghana. *Maternal and Child Nutrition*.

[14] Rikimaru T, Yartey JE, Taniguchi K, Kennedy DO, Nkrumah FK (1998). Risk factors for the prevalence of malnutrition among urban children in Ghana. *Journal of Nutritional Science and Vitaminology*.

[15] Kikafunda JK, Walker AF, Collett D, Tumwine JK (1998). Risk factors for early childhood malnutrition in Uganda. *Pediatrics*.

[16] Sakisaka K, Wakai S, Kuroiwa C (2006). Nutritional status and associated factors in children aged 0-23 months in Granada, Nicaragua. *Public Health*.

[17] Henry FJ, Briend A, Fauveau V, Huttly SRA, Yunus M, Chakraborty J (1993). Risk factors for clinical marasmus: a case-control study of Bangladeshi children. *International Journal of Epidemiology*.

[18] Odunayo SI, Oyewole AO (2006). Risk factors for malnutrition among rural Nigerian children. *Asia Pacific Journal of Clinical Nutrition*.

[19] Coulter JBS, Omer MIA, Suliman GI, Moody JB, MacFarlane SBJ, Hendrickse RG (1988). Protein-energy malnutrition in northern Sudan: prevalence, socio-economic factors and family background. *Annals of Tropical Paediatrics*.

[20] Radebe BZ, Brady P, Siziya S, Todd H (1996). Maternal risk factors for childhood malnutrition in the mazowe district of Zimbabwe. *Central African Journal of Medicine*.

[21] Getaneh T, Assefa A, Tadesse Z (1998). Protein-energy malnutrition in urban children: prevalence and determinants. *Ethiopian Medical Journal*.

[22] Wang X, Wang Y, Kang C (2005). Feeding practices in 105 counties of rural China. *Child*.

[23] Bloss E, Wainaina F, Bailey RC (2004). Prevalence and predictors of underweight, stunting, and wasting among children aged 5 and under in Western Kenya. *Journal of Tropical Pediatrics*.

[24] Beghin ID (1970). Nutritional rehabilitation centers in Latin America: a critical assessment. *American Journal of Clinical Nutrition*.

